# Emotional blunting in patients with depression. Part II: relationship with functioning, well-being, and quality of life

**DOI:** 10.1186/s12991-022-00392-4

**Published:** 2022-06-20

**Authors:** Michael Cronquist Christensen, Hongye Ren, Andrea Fagiolini

**Affiliations:** 1 Medical Affairs, H. Lundbeck A/S, Ottiliavej 9, 2500 Valby, Denmark; 2 Division of Psychiatry, Department of Molecular and Developmental Medicine, University of Siena School of Medicine, Siena, Italy

**Keywords:** Depression, Emotional blunting, Functioning, Functioning Assessment Short Test, Oxford Depression Questionnaire, Quality of life, Well-being, Patient perspectives, Recovery

## Abstract

**Background:**

Emotional blunting is a common symptom in people with depression and an important factor preventing full functional recovery. This international survey investigated the experience of emotional blunting in the acute and remission phases of depression from the perspective of patients and healthcare providers. This paper presents data on the impact of emotional blunting on overall functioning and health-related quality of life from the patient perspective.

**Methods:**

Respondents were adults diagnosed with depression by a physician, currently prescribed an antidepressant, and reporting emotional blunting during the past 6 weeks. Assessments included the Oxford Depression Questionnaire (ODQ), the Functioning Assessment Short Test (FAST), and the World Health Organization-Five Well-being Index (WHO-5). Pearson correlation and multivariate regression analyses were applied to examine the relationship between ODQ and FAST scores.

**Results:**

Data are available for 752 patients (62% female; mean age, 45 years). Mean ODQ total score was 94.8 in patients in the acute phase of depression (*n* = 300) and 85.7 in those in remission (*n* = 452; possible maximum, 130). Mean FAST total scores were 47.0 and 33.5, respectively (possible maximum, 72). Patients in the acute phase of depression had significantly greater impairment in functioning across all FAST domains than those in the remission phase (all differences, *p* < 0.01). Mean WHO-5 scores were 6.4 and 9.8 in the acute and remission phases, respectively (lower scores indicate poorer well-being). Overall, 65% of patients in the acute phase and 36% of those in remission reported that emotional blunting had a significant impact on their quality of life. Pearson correlation analysis showed a moderate positive correlation between ODQ and FAST total scores (*r* = 0.52) and a weak negative correlation between ODQ total score and WHO-5 score (*r* = − 0.26; both *p* < 0.01). In multivariate regression analysis, ODQ total score (in combination with other covariates) was the strongest significant predictor of poor patient functioning.

**Conclusions:**

Emotional blunting has a substantial negative impact on patients’ daily functioning, well-being, and quality of life in both the acute and remission phases of depression. These findings highlight the importance of recognizing and treating emotional blunting in patients with major depressive disorder in order to achieve full functional recovery.

**Supplementary Information:**

The online version contains supplementary material available at 10.1186/s12991-022-00392-4.

## Background

Depression is one of the most prevalent and disabling health conditions in adults, affecting more than 264 million people worldwide [1, 2]. The lifetime prevalence of major depressive disorder (MDD) is approximately 15–18% [3], meaning that approximately one in five people will experience a major depressive episode at some time during their life. Depression is associated with a wide range of emotional, physical, and cognitive symptoms that have a significant impact on patients’ functioning and health-related quality of life [2, 4, 5]. Functional recovery is an important goal in patients with depression [6–9]. Indeed, patients consider a return to their usual level of functioning to be one of the most important factors in defining remission from depression [10]. However, functional impairment often persists after resolution of other depressive symptoms [5]. This is of clinical significance, as residual functional impairment following remission of mood symptoms has been shown to predict subsequent relapse in patients with depression [11].

Emotional blunting is a common symptom in patients with depression, particularly those treated with selective serotonin reuptake inhibitors (SSRIs) or serotonin-noradrenaline reuptake inhibitors (SNRIs), and is increasingly recognized to be an important factor preventing full functional recovery [12–18]. Patients often report that they experience a restricted range of emotions during long-term treatment with SSRIs or SNRIs [18]. Patients may report ‘numbing’ or ‘flattening’ of emotions, emotional indifference, or reduced emotional responsiveness [15]. In a recent international survey in approximately 1400 adult antidepressant users asked about adverse effects “as a result of taking the antidepressant”, 71% of respondents reported feeling ‘emotionally numb’ and 70% reported ‘feeling foggy or detached’ [17]. Two-thirds of patients (66%) reported ‘feeling not like myself’, while 60% reported ‘reduction in positive emotions’ [17]. Emotional blunting is also a common reason for discontinuing antidepressant therapy [19]. In an online survey undertaken to identify determinants of treatment effectiveness and tolerability in patients with MDD or bipolar disorder, emotional blunting was found to be a common treatment-emergent adverse effect, leading to discontinuation of antidepressant medication in more than one-third of patients [19].

Emotional blunting is clinically important and burdensome for patients; for example, they report feeling numb, less able to laugh or cry, unable to enjoy what they used to enjoy, having less empathy, loss of inspiration or passion for creative activities, and feeling indifferent toward others [16]. It can impact on all aspects of daily living, including patients’ work, social, and family lives [12]; for example, patients experiencing emotional blunting may evade or ignore their responsibilities, which can lead to problems at work or school and to financial difficulties, as well as having a detrimental effect on their personal relationships [12]. These functional consequences of emotional blunting may be particularly relevant for patients in remission from prominent mood and anxiety symptoms, as the inability to respond to external stimuli (positive or negative) may result in premature discontinuation of treatment and thereby increased risk of relapse.

Depression is a highly heterogeneous condition, and the need to fully characterize the clinical presentation of MDD in the individual patient is increasingly recognized [7, 29, 30]. Better recognition of the most bothersome symptoms experienced by patients with MDD may permit more targeted therapeutic intervention, which may be expected to improve treatment outcomes [7, 20, 21]. However, data are lacking concerning the impact of emotional blunting on functioning, well-being, and quality of life in patients with depression.

The present study was undertaken to specifically explore the experience of emotional blunting in patients with MDD, and its impact on overall functioning and health-related quality of life, in both the acute and remission phases of depression, from the perspective of patients and healthcare providers. Our findings concerning the experience of emotional blunting from the patient perspective have been reported previously [22]. The current paper—the second in a series reporting the results of this study—presents data concerning the impact of emotional blunting on functioning, general well-being, and overall quality of life from the patient perspective. Subsequent papers in this series will report findings concerning the relationship between psychological trauma and emotional blunting and the level of concordance between patient and healthcare provider perspectives on emotional blunting in MDD.

## Methods

### Study design and participants

This was a cross-sectional, observational study conducted by BPR Pharma (London, UK) in Brazil, Canada, and Spain between April 15 and May 18, 2021. The study design has been reported in detail in the previous paper in this series [22]. In brief, data were collected through an online self-completed survey. Participants were recruited through an existing online panel of consumers and healthcare providers.

Patient respondents were aged 18–70 years and had been diagnosed with depression by a physician, were currently using a prescribed antidepressant, and reported having experienced emotional blunting in the last 6 weeks in response to a validated screening question [23]: *‘Emotional effects of depression and treatment vary, but may include, for example, feeling emotionally “numbed” or “blunted” in some way; lacking positive emotions or negative emotions; feeling detached from the world around you; or “just not caring” about things that you used to care about. Have you experienced such emotional effects during the last 6 weeks?’.*

Enrollment quotas were imposed with respect to age (50% aged ≥ 50 years) and sex (60% female). Patients who had not been diagnosed with depression by a doctor and those who responded ‘No’ to the screening question or who were not in the acute or remission phase of depression were excluded from study participation. Patients currently employed by a pharmaceutical company or market research agency were also excluded.

The study was approved by an institutional review board (Veritas IRB, Montreal, QC, Canada), was conducted in accordance with the European Pharmaceutical Market Research Association (EphMRA) code of conduct, and adhered to General Data Protection Regulation (GDPR) and all local market laws regarding data protection. Patients had previously consented to participate in research; however, informed consent was also obtained specifically for this study.

### Outcome measures

The severity of emotional blunting was assessed using the Oxford Depression Questionnaire (ODQ) [23, 24]. The ODQ comprises 26 questions about emotional experiences during the past week, for which respondents are asked the extent to which they agree or disagree. Questions are split across five domains: general reduction in emotions, reduction in positive emotions, emotional detachment from others, not caring, and antidepressant as cause. The ‘antidepressant as cause’ domain is only completed by patients who are currently taking antidepressants, and explores the perception of a potential link between the patient’s current treatment and their experience of emotional blunting. The ODQ total score ranges from 26 to 130 points, with higher scores indicating more severe emotional symptoms.

Functioning was assessed by means of the Functioning Assessment Short Test (FAST), a brief self-report instrument designed to assess problems experienced in daily functioning [25]. The FAST covers 24 items across six domains of functioning: autonomy, occupational functioning, cognitive functioning, financial issues, interpersonal relationships, and leisure time. Patients were instructed to select the degree of difficulty associated with each item (‘no difficulty’, ‘mild difficulty’, ‘moderate difficulty’, ‘severe difficulty’, or ‘don’t know’). For the purposes of this survey, the period of recall was ‘*during this acute or remission phase of depression*.’ FAST total score ranges from 0 to 72, with higher scores indicating greater functional impairment.

General well-being was assessed using the World Health Organization-Five Well-being Index (WHO-5) [26]. This short self-reported questionnaire assesses the degree of agreement over the past 2 weeks with five simple statements covering different aspects of well-being; agreement scores range from 0 (at no time) to 5 (all of the time). WHO-5 total score ranges from 0 to 25. Higher scores indicate a greater sense of well-being, with a total score of less than 13 considered indicative of poor well-being.

Patients were asked which of a list of symptoms they had experienced during their current phase of depression (including emotional blunting), and were asked to rate the severity of the symptoms experienced on a scale of 1 (not at all severe) to 7 (extremely severe). Patients also rated the impact of their symptoms [namely, emotional blunting, lack of interest (i.e., anhedonia), mood symptoms, physical symptoms, anxiety, and cognitive symptoms] on different aspects of daily life; namely, their ability to function at work, in their home/family life, and in their social life, as well as on their overall quality of life. Impact was rated on a 7-point scale, with a score of 6 or 7 indicating a significant impact.

### Statistical analysis

The analysis population comprised all respondents who met the inclusion criteria and completed the online survey. Patients who failed to complete the survey or who completed the survey much faster than average were excluded from the final sample. Any patient who responded ‘don’t know’ to more than one item for any FAST domain was excluded from the analysis of that domain. For patients who responded ‘don’t know’ to just one item in any FAST domain, the mean score for the other items answered in that domain was used to impute the missing value (as per the scale guidance). Respondents with a missing score for any domain were removed from the calculation of FAST total score (a missing score would result from them having responded ‘don’t know’ to more than one item in that domain).

Data were analyzed for the overall patient population and by phase of depression (self-reported): acute or remission. The acute phase was defined as: *‘A time when your symptoms are at their worst or most severe and for which you use antidepressant treatment.’* The remission phase was defined as: ‘*A time when your symptoms have improved significantly and you are already feeling better, but you may or may not still experience some minor symptoms. You are still taking antidepressant medication.’*

Results are presented descriptively using means and standard deviations (SDs) for continuous variables, and frequencies and percentages for categorical variables. Comparisons were performed for continuous measures using *t* tests and for proportions using *Z* tests. The relationship between ODQ total score and FAST and WHO-5 total scores was explored using Pearson correlation and multivariate regression analyses. Regression was assessed on: (a) demographic variables (age band, sex, education, country); (b) symptoms of depression (anxiety, physical symptoms, cognitive symptoms, mood symptoms); and (c) ODQ total score. Variable sets (a), (b), and (c) were entered hierarchically as blocks. Block (a) was force entered, and variables in blocks (b) and (c) were entered only if they had a statistically significant effect (determined using backward and forward selection). Statistical significance determined by either method was considered valid. For FAST total score, the analysis was also performed using the five ODQ domain scores instead of the ODQ total score.

Data were analyzed by The Stats People (Sevenoaks, UK) using MERLIN tabulation software and Microsoft Excel. Significance was set at *p* < 0.05.

## Results

### Clinical characteristics

Data were available for 752 patients (300 in the acute phase of depression and 452 in remission). In all, 120 patients were excluded from the calculation of FAST scores. Patient demographics are summarized in Table 1. Mean (SD) age was 45 (12) years and 62% of respondents were female. Most patients were receiving treatment with an SSRI or SNRI, most commonly fluoxetine (received by 26% of all patients). The overall mean (SD) ODQ total score was 89.3 (18.3) points (possible maximum, 130 points), indicative of severe emotional blunting. Mean (SD) ODQ total score was significantly higher in patients in the acute phase of depression than in those in the remission phase (difference between depression phases, *p* < 0.01). There was no difference in severity of emotional blunting (i.e., mean ODQ total score) between countries (Additional file 1: Table S1).

**Table 1:** Patient demographics and clinical characteristics, overall and according to phase of depression

	All patients (*N* = 752)	Acute (*n* = 300)	Remission (*n* = 452)
Sex, *n* (%)			
Female	466 (62)^a^	195 (65)^a^	271 (60)^a^
Age, years			
Mean (SD)	45 (12)	45 (12)	46 (13)
Time since diagnosis of depression (months)			
Mean (SD)	62.4 (49.5)	61.3 (48.3)	63.2 (50.3)
High school education or above, *n* (%)	596 (79)	235 (78)	361 (80)
Work status, *n* (%)			
Full-time	348 (46)	120 (40)	228 (50)**
Part-time	93 (12)	33 (11)	60 (13)
In a relationship, *n* (%)	471 (63)	181 (60)	290 (64)
Current antidepressant treatment, *n* (%)^b^			
Fluoxetine	199 (26)	87 (29)	112 (25)
Escitalopram	125 (17)	52 (17)	73 (16)
Sertraline	121 (16)	53 (18)	68 (15)
Citalopram	110 (15)	48 (16)	62 (14)
Venlafaxine	100 (13)	46 (15)	54 (12)
Bupropion	79 (11)	46 (15)**	33 (7)
Paroxetine	78 (10)	32 (11)	46 (10)
Clinical assessment scores, mean (SD)			
ODQ total score	89.3 (18.3)	94.8 (16.6)**	85.7 (18.4)
FAST total score	38.7 (17.3)	47.0 (15.8)**	33.5 (16.1)
WHO-5 total score	8.5 (5.5)	6.4 (5.6)**	9.8 (5.0)

*FAST* Functioning Assessment Short Test, *ODQ* Oxford Depression Questionnaire, *SD* standard deviation, *WHO-5* World Health Organization-Five Well-being Index

Difference between acute and remission phases, **p < 0.01

^a^A quota of 60% female was set in the study design

^b^Only antidepressants received by ≥ 10% of patients in either phase of depression are shown; patients may have received more than one drug therapy

**Fig. 1:**
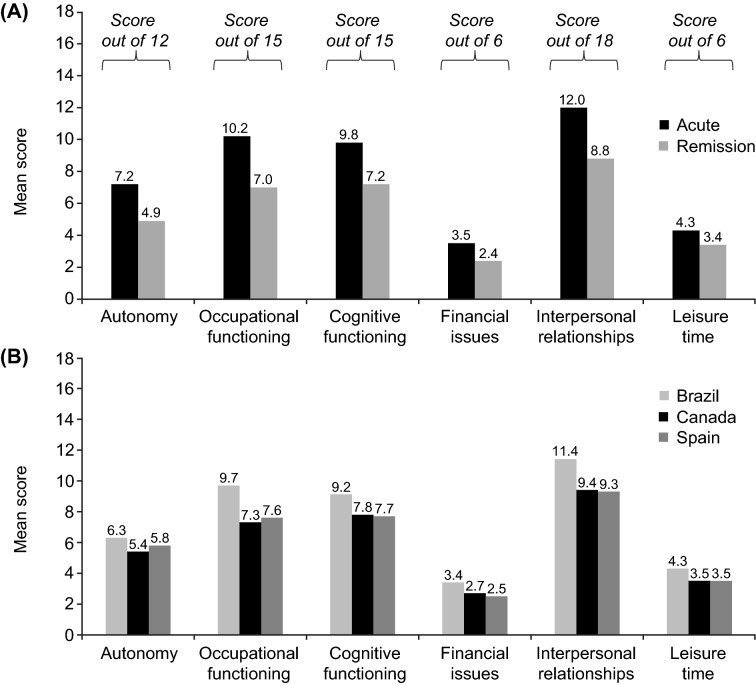
Mean patient-reported FAST domain scores **A** by phase of depression (acute, *n* = 252–299; remission, *n* = 398–452 across domains), and **B** by country (Brazil, *n* = 223–251; Canada, *n* = 207–250; Spain, *n* = 220–250). *p* < 0.01 for all differences between the acute and remission phases and all differences between Brazil and other countries, with the exception of the autonomy domain (Brazil vs Canada, *p* < 0.01; Brazil vs Spain, not significant). *FAST* Functioning Assessment Short Test

**Fig. 2:**
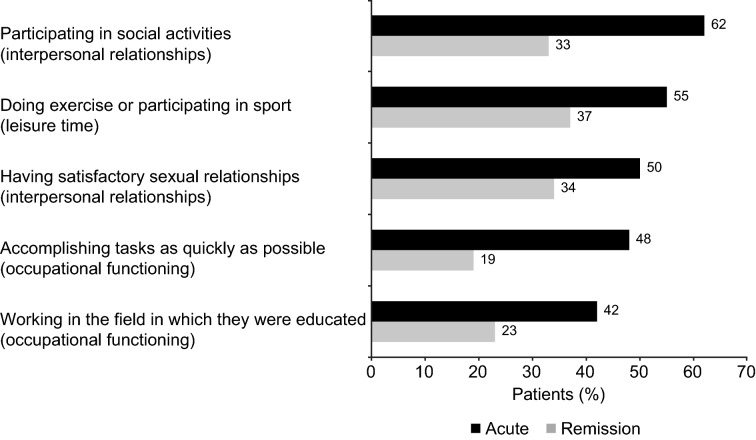
The top five individual FAST items that the patients report greatest difficulty with by phase of depression (acute, *n* = 300; remission, *n* = 452). The proportion of patients reporting severe difficulty with each item is shown. *p* < 0.01 for all differences between the acute and remission phases. *FAST* Functioning Assessment Short Test

The overall mean (SD) FAST total score was 38.7 (17.3) points (possible maximum, 72 points). Mean (SD) FAST total score was significantly higher in the acute phase of depression than in the remission phase (difference, *p* < 0.01). Patients in the acute phase had significantly greater impairment in functioning across all FAST domains than those in remission (Fig. 1; *p* < 0.01 for all differences between depression phases). Irrespective of the phase of depression, the greatest impairment was seen in the ‘interpersonal relationships’, ‘occupational functioning’, and ‘cognitive functioning’ domains. The five individual FAST items with which patients reported greatest difficulty were participating in social activities (reported by 45% of patients), doing exercise or participating in sport (44%), having satisfactory sexual relationships (41%), accomplishing tasks as quickly as possible (31%), and working in the field in which they were educated (31%). For all these items, patients in the acute phase of depression were significantly more likely than those in remission to report severe difficulty (Fig. 2). Mean FAST total score was significantly higher in patients from Brazil than in those from Canada or Spain (both differences, *p* < 0.01) (Additional file 1: Table S1). Across all countries, the greatest impairment was seen in the ‘interpersonal relationships’, ‘cognitive functioning’, and ‘occupational functioning’ domains (Fig. 1).

The mean (SD) WHO-5 total score was 8.5 (5.5) points, indicating poor general well-being (i.e., WHO-5 total score < 13). Overall, 25% of patients said that at no time during the past 2 weeks had they felt active and vigorous, and 30% said that at no time had they woken up feeling fresh and rested. Patients reported poor well-being in both the acute and remission phases of depression; however, well-being was significantly worse in patients in the acute phase (difference between depression phases, *p* < 0.01). No differences in mean WHO-5 total scores were observed between countries (Additional file 1: Table S1).

### Impact of symptoms on functioning and overall quality of life

**Fig. 3:**
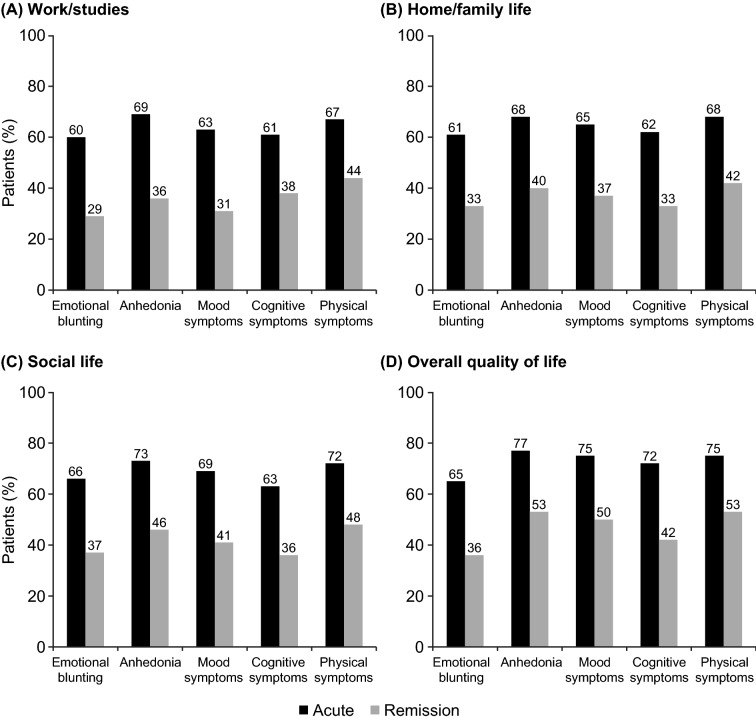
Proportion of patients reporting a significant impact of emotional blunting, anhedonia, mood symptoms, cognitive symptoms, and physical symptoms on **A** work/studies, **B** home/family life, **C** social life, and **D** overall quality of life by phase of depression (acute, *n* = 300; remission, *n* = 452). *p* < 0.01 for all differences between acute and remission phases

The impact of symptoms of depression on functioning and overall quality of life is summarized in Fig. 3. A significant impact of emotional blunting on functioning in their work, home, and social life was reported by 60–66% of patients in the acute phase of depression and 29–37% of those in remission (*p* < 0.01 for all differences between depression phases). In all, 65% of patients in the acute phase of depression and 36% of those in remission reported that emotional blunting had a significant impact on their overall quality of life (*p* < 0.01).

A significant impact of anhedonia on functioning and overall quality of life was reported by 68–77% of patients in the acute phase of depression and 36–53% of those in remission (*p* < 0.01 for all differences between depression phases). Corresponding proportions of patients reporting a significant impact on functioning and quality of life were 63–75% versus 31–50% for mood symptoms, 61–72% versus 33–42% for cognitive symptoms, and 67–75% versus 42–53% for physical symptoms (i.e., fatigue or lack of energy) (*p* < 0.01 for all differences between depression phases).

### Association analyses

In the Pearson correlation analysis, significant correlations were seen between ODQ total score and FAST and WHO-5 total scores (Table 2). A moderate positive correlation was seen between ODQ total score and FAST total score and a weak negative correlation between ODQ total score and WHO-5 total score (both *p* < 0.01). Significant positive correlations were seen between all ODQ domains and FAST total score (all *p* < 0.01); the strongest correlations were seen with the ‘not caring’ and ‘reduction in positive emotions’ domains (both *p* < 0.01). For WHO-5 total score, significant correlations were only observed for the ‘reduction in positive emotions’ and ‘not caring’ domains of the ODQ (both *p* < 0.01).

**Table 2:** Analysis of correlations between ODQ total and domain scores and FAST and WHO-5 total scores.

	FAST total score (*N* = 632)	WHO-5 total score (*N* = 752)
ODQ total score	0.52*	− 0.26*
General reduction in emotions	0.23*	− 0.07
Reduction in positive emotions	0.46*	− 0.47*
Emotional detachment from others	0.36*	− 0.05
Not caring	0.58*	− 0.27*
Antidepressant as cause	0.27*	0.06

Pearson correlation coefficients are shown

*FAST* Functioning Assessment Short Test, *ODQ* Oxford Depression Questionnaire, *WHO-5* World Health Organization-Five Well-being Index

^*^Correlation is significant at 0.01 level (two-tailed)

**Fig. 4:**
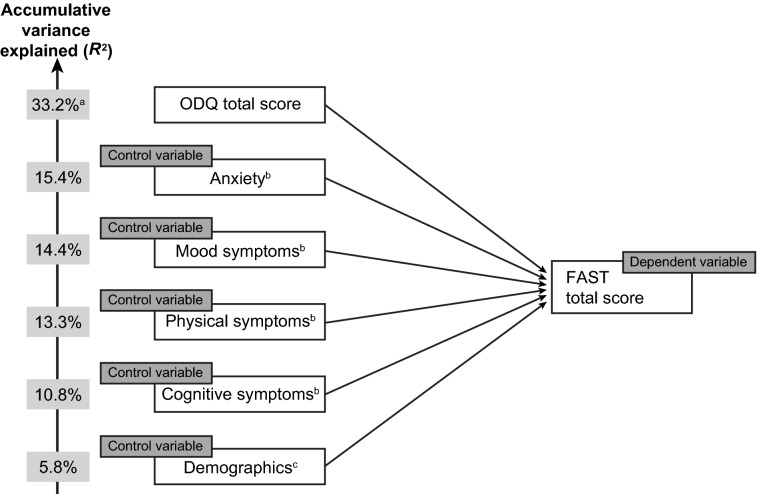
Stepwise multivariate regression analysis showing the extent to which FAST total score is predicted by ODQ total score. ^a^ODQ percentage is accumulative, reflecting ODQ and control variables, ^b^anxiety, mood symptoms (sadness, lack of enjoyment, hopelessness), physical symptoms (decrease in weight or appetite, disturbed sleep, fatigue, sexual dysfunction), and cognitive symptoms (trouble concentrating, difficulties making plans, forgetfulness) were assessed by the survey questionnaire, ^c^age, sex, education, and country. *FAST* Functioning Assessment Short Test, *ODQ* Oxford Depression Questionnaire.

In the multivariate regression analysis, ODQ total score (in combination with control variables) was found to be the strongest significant predictor of FAST total score, accounting for over half of the 33.2% accumulative variance in FAST total score observed (Fig. 4). Holding all other variables equal, a 10-point increase in ODQ total score led to a 4.8-point increase in FAST total score. After emotional blunting (i.e., ODQ total score), physical symptoms and cognitive symptoms were found to have a larger effect on FAST total score than other depressive symptoms; demographic characteristics only weakly predicted FAST total score. In the model containing the individual ODQ domains, the ‘not caring’ domain was found to be the dominant predictor of FAST total score after controlling for demographics and symptoms. Holding all other variables equal, an increase in ‘general reduction in emotions’ was associated with a decrease (i.e., improvement) in FAST total score.

**Fig. 5:**
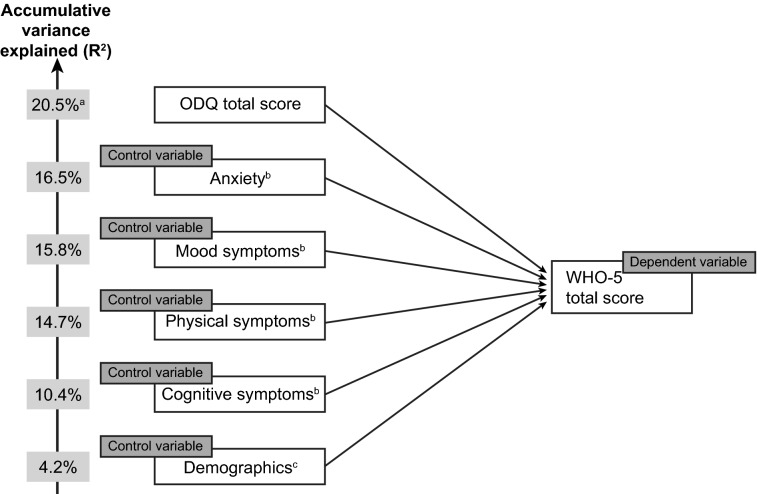
Stepwise multivariate regression analysis showing the extent to which WHO-5 total score is predicted by ODQ total score. ^a^ODQ percentage is accumulative, reflecting ODQ and control variables, ^b^anxiety, mood symptoms (sadness, lack of enjoyment, hopelessness), physical symptoms (decrease in weight or appetite, disturbed sleep, fatigue, sexual dysfunction), and cognitive symptoms (trouble concentrating, difficulties making plans, forgetfulness) were assessed by the survey questionnaire, ^c^age, sex, education, and country. *ODQ* Oxford Depression Questionnaire, *WHO-5* World Health Organization-Five Well-being Index

Cognitive symptoms were found to have the greatest impact on WHO-5 total score. ODQ total score (in combination with control variables) was found to account for approximately one-fifth of the overall accumulative variance in WHO-5 total score (total *R*-squared, 20.5%) (Fig. 5). Holding all else equal, a 10-point increase in ODQ total score led to a 0.8-point decrease (i.e., worsening) in WHO-5 total score.

## Discussion

Results of this study highlight the substantial negative impact of emotional blunting on all aspects of patients’ daily functioning, well-being, and health-related quality of life in both the acute and remission phases of depression. The negative impact of emotional blunting on patient functioning was greater than that of all other symptoms of depression, including mood symptoms, anxiety, cognitive symptoms, and physical symptoms. As might be expected, functioning was found to be more negatively impacted in patients in the acute phase of depression than in those in remission across all individual FAST domains (autonomy, occupational functioning, cognitive functioning, financial issues, interpersonal relationships, and leisure time). The impact of emotional blunting on patient’s overall well-being and quality of life was also more pronounced in patients in the acute phase of the disease. Nevertheless, approximately one-third of patients who were in remission from their core symptoms of depression still reported a significant negative impact of emotional blunting on their daily functioning, general well-being, and quality of life.

The FAST total scores seen in this study are similar to those reported in a previous multinational survey of more than 2000 patients with MDD that was undertaken to assess differences in perceptions of symptoms of depression and treatment priorities between patients and healthcare providers across the different phases of depression [27]. In that survey, the overall mean FAST total score was 40.5 points (46.7 in patients in the acute phase of depression and 33.4 in those in remission). As in the present study, patients reported greatest impairment in the interpersonal relationship, cognitive functioning, and occupational functioning domains.

In the present study, patients with depression experiencing emotional blunting reported a marked impairment in general well-being, irrespective of their phase of depression (WHO-5 total score, 6.4 in patients in the acute phase of depression and 9.8 in those in remission). These WHO-5 total scores are lower (i.e., worse) than that previously reported in patients with moderately severe depression (i.e., Montgomery Åsberg Depression Rating Scale total score ≥ 20) who were initiating antidepressant treatment (WHO-5 total score, 14.8 points) [28]. The WHO-5 total score in patients in the acute phase of depression in the present study is very similar to that reported at baseline in a pooled analysis of data from pivotal studies of desvenlafaxine (5.9 points) [29]; however, the WHO-5 total score in patients in remission is lower than that reported in the pooled analysis population at study end (12.7 points) [29]. A difference of 2.5 points in WHO-5 total score has been suggested to be clinically relevant in patients with depression [29]. Collectively, these data suggest that emotional blunting may have the greatest impact on overall well-being in patients with MDD during the remission phase of the disease.

In the stepwise multivariate analysis, emotional blunting (i.e., ODQ total score) was found to be the strongest predictor of impaired functioning, accounting for a greater proportion of the overall accumulative variance in FAST total score than demographic characteristics and other depressive symptoms (i.e., anxiety and mood, cognitive, and physical symptoms). In light of the existing literature concerning the impact of individual symptoms of depression on patient functioning, this is quite a remarkable finding. For example, an analysis of data from the landmark Sequenced Treatment Alternatives to Relieve Depression (STAR*D) study of 3703 outpatients with depression receiving their first antidepressant treatment found sad mood and problems with concentration to be the most debilitating symptoms in terms of impact on patient functioning (assessed using the Work and Social Functioning Scale) [30].

The observed relationship between emotional blunting and both functioning and health-related quality of life in the present study suggests a clinical need for improved recognition and treatment of this symptom in both the acute and remission phases of depression if patients are to achieve full functional recovery. The simple, validated screening question for emotional blunting from the ODQ could readily be used in routine practice settings to facilitate identification of patients with depression who are experiencing emotional blunting, whether as a symptom of MDD or as an adverse effect of their current antidepressant medication. As previously reported, almost half of the patients in the cohort enrolled in the present study believed that their antidepressant medication was affecting their emotions and just over one-third were considering stopping or had already stopped taking their prescribed antidepressant because of treatment-emergent emotion-related side effects [22]. Patients experiencing emotional blunting on their current antidepressant may benefit from switching to an alternative drug for depression.

Prevention of relapse is particularly important in the remission phase of the disease, as patients return to work and resume normal social activities. Patients with remitted MDD who continue to experience functional impairment have been shown to be more likely to subsequently relapse [11]. In a recent study in patients with depression with an inadequate response and emotional blunting after SSRI or SNRI therapy, the significant improvement in emotional blunting observed during subsequent treatment with vortioxetine was found to be strongly and significantly correlated with improvements in overall functioning, as well as motivation and energy [31]. These associations persisted even after adjustment for improvement in depressive symptom severity. Mediation analysis showed that almost two-thirds of the improvement in functioning was a direct effect of the improvement in emotional blunting seen after switching to vortioxetine [31]. In another analysis, improvements in functioning in patients with MDD in placebo-controlled studies of vortioxetine were shown to be mostly driven by the beneficial effect of treatment on symptoms of anhedonia [32]. Anhedonia (i.e., the inability to experience pleasure) is an aspect of emotional blunting that is recognized as one of the core diagnostic criteria for a major depressive episode [33].

## Methodological considerations

Study strengths and limitations have been described in detail in the previous paper in this series [19]. In brief, the main strength of this study is that it provides information on the lived experience of emotional blunting in depression from the patient’s own perspective in a large sample recruited from different countries, and in both the acute and remission phases of the disease. To our knowledge, this is one of the most detailed assessments of emotional blunting in patients with depression undertaken to date. The internet-based survey methods used facilitated the collection of data across a large number of variables, providing a comprehensive overview of both the clinical presentation of emotional blunting in MDD and its impact on patient functioning, well-being and health-related quality of life. Possible limitations include the potential for selection and/or recall bias, lack of information concerning comorbid mental health conditions that might have influenced patient responses, and that the patient experience of emotional blunting may have been influenced by the antidepressant treatment(s) received. Furthermore, due to the cross-sectional study design, any causal inferences should be interpreted with caution.

## Conclusion

In summary, in patients with depression and emotional blunting, emotional blunting has a substantial negative impact on patients’ daily functioning, general well-being, and quality of life in both the acute and remission phases of the disease. Adjusting for the effect of demographic characteristics and other core symptoms of depression (i.e., mood, anxiety, cognitive, and physical symptoms), emotional blunting was found to be an extremely strong predictor of poor patient functioning. These findings highlight the importance of recognizing and treating emotional blunting in patients with MDD in order to help them to achieve full functional recovery and return to their normal daily lives. This could be achieved in routine practice settings using the simple, validated screening question from the ODQ.

## Supplementary Information

**Additional file 1: Table S1.** Clinical assessment scores for the patient-reported cohort by country.

## Data Availability

The datasets presented in this article are not readily available given the informed consent provided by survey participants. Requests to access the datasets should be directed to the authors.
