# Supplementary material for: Emotional blunting in patients with depression. Part II: relationship with functioning, well-being, and quality of life
Source: Ann Gen Psychiatry. 2022 Jun 20;21:20. doi: 10.1186/s12991-022-00392-4 (PMC9210577; doi:10.1186/s12991-022-00392-4)
Supplement: Supplementary file 1 — Additional file 1: Table S1. Clinical assessment scores for the patient-reported cohort by country. [file 12991_2022_392_MOESM1_ESM.pdf]

Additional file 1

Table S1 Clinical assessment scores for the patient-reported cohort by country.

**Clinical assessment scores, mean (SD)**	**Brazil (*n* = 251)**	**Canada (*n* = 251)**	**Spain (*n* = 250)**
ODQ total score	89.9 (18.3)	88.4 (18.4)	89.6 (18.2)
FAST total score	44.2 (16.7)**	35.9 (16.0)	35.7 (17.8)
WHO-5 total score	8.2 (5.3)	8.8 (5.8)	8.4 (5.4)

Difference versus Canada and Spain, **$p < 0.01$.

FAST, Functioning Assessment Short Test; ODQ, Oxford Depression Questionnaire, SD, standard deviation; WHO-5, World Health Organization-Five Well-Being Index.
